# *Mycobacterium bovis* Infection in African Wild Dogs, Kruger National Park, South Africa

**DOI:** 10.3201/eid2507.181653

**Published:** 2019-07

**Authors:** Roxanne L. Higgitt, O. Louis van Schalkwyk, Lin-Mari de Klerk-Lorist, Peter E. Buss, Peter Caldwell, Leana Rossouw, Tebogo Manamela, Guy A. Hausler, Jennie Hewlett, Emily P. Mitchell, Paul D. van Helden, Sven D.C. Parsons, Michele A. Miller

**Affiliations:** Stellenbosch University, Cape Town, South Africa (R.L. Higgitt, G.A. Hausler, P.D. van Helden, S.D.C. Parsons, M.A. Miller);; Department of Agriculture, Forestry and Fisheries, Skukuza, South Africa (O.L. van Schalkwyk, L.-M. de Klerk-Lorist);; South African National Parks, Skukuza (P.E. Buss, L. Rossouw, T. Manamela, J. Hewlett);; Old Chapel Veterinary Clinic, Pretoria, South Africa (P. Caldwell);; National Zoological Gardens of South Africa, Pretoria (E.P. Mitchell)

**Keywords:** African wild dog, bovine tuberculosis, conservation, infection prevalence, interferon-gamma release assay, Mycobacterium bovis, bacteria, South Africa, tuberculosis and other mycobacteria

## Abstract

We screened African wild dogs (*Lycaon pictus*) in Kruger National Park, South Africa, for *Mycobacterium bovis* infection using an interferon-gamma release assay. We detected *M. bovis* sensitization in 20 of 21 packs; overall apparent infection prevalence was 83%. These animals experience high infection pressure, which may affect long-term survival and conservation strategies.

The African wild dog (*Lycaon pictus*) is an endangered carnivore occurring in fragmented, small populations (in South Africa, <500 animals). These factors make them susceptible to adverse factors, such as infectious diseases, that may threaten their long-term survival (*1**,**2*). Of particular concern are diseases caused by multihost pathogens that are capable of persisting in reservoir host species, such as *Mycobacterium bovis*, the causative agent of bovine tuberculosis (bTB). This pathogen may pose a major threat to the conservation of endangered host populations (*3*).

Since 2012, sporadic cases of wild dogs with macroscopic and histological lesions consistent with tuberculosis (TB) have been recorded in South Africa, specifically in Kruger National Park (KNP; n = 8), uMkuze Game Reserve (n = 1), and Hluhluwe-iMfolozi Park (HiP; n = 2). *M. bovis* infection is endemic in these parks and occurs in multiple species that are preyed upon by wild dogs, such as warthogs, which have an estimated *M. bovis* seroprevalence up to 38% in KNP (*4**,**5*). In 2 cases from KNP, acid-fast bacilli were associated with granulomatous lymphadenitis, and spoligotype analysis of *M. bovis* isolates from lesions in affected wild dogs from KNP (strain type SB0121) and HiP (strain type SB0130) were the same as those found in local prey (*6*).

*M. bovis* is a novel pathogen of wild dogs; understanding the impact of bTB disease in wild dogs is imperative to making informed management decisions regarding these animals’ conservation. Estimation of prevalence would provide a starting point for this investigation but requires diagnostic tools for accurate detection of *M. bovis* infection. To estimate prevalence in the KNP wild dog population, we assessed sensitization to TB antigens ESAT-6 and CFP-10.

During July 2016–January 2018, we tested blood samples from 77 wild dogs from KNP using an interferon-gamma release assay (IGRA) developed by our group (*7*). We tested animals from 21 wild dog packs; 20 of these included >1 IGRA-positive animal, indicating widespread exposure to *M. bovis* throughout KNP (Figure). We observed no significant difference in IGRA results based on sex (p = 0.79 by 2-tailed Mann-Whitney test). Overall, the apparent prevalence of *M. bovis* infection was 82% (63/77; 95% CI 72%–89% by modified Wald test).

**Figure:**
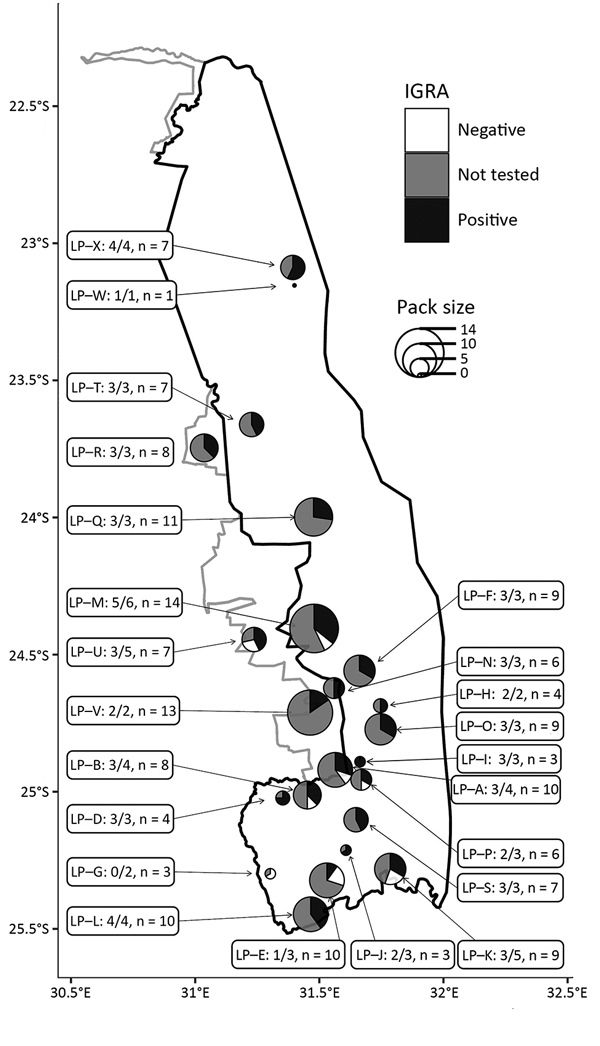
*Mycobacterium bovis* infection in African wild dog packs, Kruger National Park, South Africa. Each pie chart indicates the position of a pack at time of sampling; the size of the pack (n value); and the proportion of test-positive, test-negative, and untested animals. The pack name (e.g., LP-A) and the proportion of tested animals that were test-positive (e.g., 2/2) are shown. A single wild dog that was not part of a pack was included. IGRA, interferon gamma release assay.

Few reports of active bTB disease and related deaths have been documented in wild dogs, so the high apparent prevalence of *M. bovis* infection in the KNP population is surprising. However, similarly high prevalence estimates have been reported for African lions (*Panthera leo leo*) in KNP using the tuberculin skin test (55%; 95% CI 45%–65%) (*8*) and a cytokine gene expression assay (44%; 95% CI 32%–57%) (*9*). These results highlight the high infection pressure for carnivores within KNP.

The prevalence estimate in wild dogs was based on detection of immune sensitization to *M. bovis*–specific antigens. Although the infection status of a small number of animals was confirmed by antemortem mycobacterial culture of oropharyngeal swabs (4 [7.0%] of 57 wild dogs tested), we could not confirm the infection status of all of the animals included in this study. Furthermore, during the time of the study, most animals included in this analysis appeared to be healthy. Therefore, further investigations will be required to clarify the progression of *M. bovis* infection and risk of bTB disease in this species.

These results have implications for managing the wild dog metapopulation in South Africa, which involves translocation of animals across the country to maintain genetic diversity and to achieve conservation goals (*2*). The risk of introducing *M. bovis* into previously uninfected areas by an infected wild dog is unknown, and studies on transmission will be crucial in assessing this risk.

Survival of a species is affected by a complex array of factors, of which disease is only one. Currently, the KNP wild dog population appears to be stable (*2*), despite the apparent high prevalence of *M. bovis* infection. Favorable conditions, such as abundance of prey, may support high reproductive rates. However, with environmental changes, such as prolonged drought, the vulnerability of host populations to infectious disease may be more pronounced (*10*).

In conclusion, this study shows widespread exposure of wild dog packs to *M. bovis* in KNP, with high infection pressure to individual dogs. Although the impact of disease on population numbers is unknown, wild dogs infected with *M. bovis*, even young animals, have been observed to have generalized disease leading to death. Further investigations into the progression of *M. bovis* infection, the risk for transmission, and the probability of developing progressive disease are needed to assess the threat of this emerging disease in African wild dog populations.
